# Validation of the English version of the UNESP-Botucatu multidimensional composite pain scale for assessing postoperative pain in cats

**DOI:** 10.1186/1746-6148-9-143

**Published:** 2013-07-17

**Authors:** Juliana T Brondani, Khursheed R Mama, Stelio P L Luna, Bonnie D Wright, Sirirat Niyom, Jennifer Ambrosio, Pamela R Vogel, Carlos R Padovani

**Affiliations:** 1 Department of Veterinary Surgery and Anesthesiology, School of Veterinary Medicine and Animal Science, and the Department of Biostatistics, Institute of Biosciences, UNESP Univ Estadual Paulista, Botucatu, SP 18618-970, Brazil; 2 Department of Clinical Sciences, Colorado State University, Fort Collins, CO 80526, USA

**Keywords:** Cats, Validation, Scale, Pain, Postoperative, Ovariohysterectomy, Validity, Reliability, Responsiveness

## Abstract

**Background:**

A scale validated in one language is not automatically valid in another language or culture. The purpose of this study was to validate the English version of the UNESP-Botucatu multidimensional composite pain scale (MCPS) to assess postoperative pain in cats. The English version was developed using translation, back-translation, and review by individuals with expertise in feline pain management. In sequence, validity and reliability tests were performed.

**Results:**

Of the three domains identified by factor analysis, the internal consistency was excellent for ‘pain expression’ and ‘psychomotor change’ (0.86 and 0.87) but not for ‘physiological variables’ (0.28). Relevant changes in pain scores at clinically distinct time points (e.g., post-surgery, post-analgesic therapy), confirmed the construct validity and responsiveness (Wilcoxon test, p < 0.001). Favorable correlation with the IVAS scores (p < 0.001) and moderate to very good agreement between blinded observers and ‘gold standard’ evaluations, supported criterion validity. The cut-off point for rescue analgesia was > 7 (range 0–30 points) with 96.5% sensitivity and 99.5% specificity.

**Conclusions:**

The English version of the UNESP-Botucatu-MCPS is a valid, reliable and responsive instrument for assessing acute pain in cats undergoing ovariohysterectomy, when used by anesthesiologists or anesthesia technicians. The cut-off point for rescue analgesia provides an additional tool for guiding analgesic therapy.

## Background

The importance of using standardized and validated pain assessment tools has received recent attention [1]. One reason is that tools and techniques with established validity and reliability produce more consistent and accurate results. Another is that these validated tools/techniques enable the comparison of outcomes from different studies. For this to occur, however, it is important that these tools (e.g., pain assessment scales) are available and validated for different languages and cultures.

An instrument that has been previously validated in one language is not automatically valid in another language and culture [2-5]. Therefore simple literal translation is not appropriate but rather rigorous methodology must be followed to validate the instrument for different circumstances of language and/or culture. This ensures that the meaning and intent of the original items are maintained and that the scale remains relevant [6]. As part of this process it is suggested that the validation of the tool or scale should be performed using recognized statistical methods in the target language and/or culture [3,4].

In this context, the validation of an instrument refers to the assessment of validity and reliability. Reliability of a scale is initially be assessed by testing its internal consistency, but it is then also necessary to assess the ability of the instrument to produce similar results when used by different individuals or when used at different times by the same individual [4]. Validity is defined as the effectiveness with which a test or scale measures the property under investigation [7]. Albeit there has been some discussion pertaining to this recently, traditionally validity has been separated into three distinct aspects, namely content, criterion and construct [8,9]. The validation of a tool should focus primarily on the logic and methodology of hypothesis testing, and the distinct concepts aforementioned should be preserved merely to refer to different types of validity testing [4].

The McGill pain questionnaire is one of the most commonly used tools to assess pain in man, and its translation, cultural adaptation and validation have been accomplished in different languages and cultures [10-14]. In veterinary medicine it is only recently that cross cultural use of pain scales has aroused interest; the use of the Glasgow composite measure pain scale used for assessing acute pain in dogs was recently evaluated in a different clinical environment where English was not the first language [15].

The validity and reliability of the UNESP-Botucatu-MCPS for assessing postoperative acute pain in cats has been established in its original language, Brazilian Portuguese. The scale was initially submitted to rigorous refinement [16], followed by verification of content, construct and criterion validity, inter and intra-rater reliability, responsiveness and the definition of a cut-off point for intervention analgesia [17,18].

By virtue of positive results of the validation of the scale in Brazilian Portuguese, and due to the absence of validated tools to assess acute pain in cats, the aim of this study was to validate the English version of the UNESP-Botucatu-MCPS. The hypothesis of this study was that if the translation and cultural adaptation were adequate, the English version would demonstrate reliability and validity similar to the original Brazilian Portuguese scale.

## Results

### Content validity - analysis by a committee of experts

All items of the scale, except for arterial blood pressure, showed values greater than 0.5. However, after analyzing the results, the researchers decided not to delete the item arterial blood pressure from the scale because there was no agreement among experts regarding the relevance of this item. One expert felt arterial blood pressure was relatively valid, another that it was relatively invalid and the third expert wasn’t sure of the significance. Following their review, experts suggested additional minor changes in content and organization. At their suggestion each item of the scale was standardized to have four descriptive levels. Additionally the item previously termed *mental status* was renamed *attitude*. The final scale included ten items: *posture*, *comfort*, *activity*, *attitude*, *miscellaneous behaviors*, *reaction to palpation of the surgical wound*, *reaction to palpation of the abdomen/flank*, *arterial blood pressure*, *appetite* and *vocalization*. Each item was assigned a score of 0 – 3 with 0 indicating normal or no change and 3 indicating the most marked change for the item. The total score, calculated from the sum of the item scores thus ranged from 0 (arbitrary absence of pain) to 30 (maximum pain) (Table 1).

**Table 1:** The English version of the UNESP-Botucatu-MCPS after content analysis and rearrangement of domains

	**Subscale 1: Pain expression (0 – 12)**	
**Miscellaneous behaviors**	Observe and mark the presence of the behaviors listed below	
	**A** - The cat is laying down and quiet, but moving its tail	A
	**B** - The cat contracts and extends its pelvic limbs and/or contracts its abdominal muscles (flank)	B
	**C** - The cats eyes are partially closed (eyes half closed)	C
	**D** - The cat licks and/or bites the surgical wound	D
	• All above behaviors are absent	0
	• Presence of one of the above behaviors	1
	• Presence of two of the above behaviors	2
	• Presence of three or all of the above behaviors	3
**Reaction to palpation of the surgical wound**	• The cat does not react when the surgical wound is touched or pressed; or no change from pre-surgical response (if basal evaluation was made)	0
	• The cat does not react when the surgical wound is touched, but does react when it is pressed. It may vocalize and/or try to bite	1
	• The cat reacts when the surgical wound is touched and when pressed. It may vocalize and/or try to bite	2
	• The cat reacts when the observer approaches the surgical wound. It may vocalize and/or try to bite. The cat does not allow palpation of the surgical wound	3
**Reaction to palpation** **of the abdomen/flank**	• The cat does not react when the abdomen/flank is touched or pressed; or no change from pre-surgical response (if basal evaluation was made). The abdomen/flank is not tense	0
	• The cat does not react when the abdomen/flank is touched, but does react when it is pressed. The abdomen/flank is tense	1
	• The cat reacts when the abdomen/flank is touched and when pressed. The abdomen/flank is tense	2
	• The cat reacts when the observer approaches the abdomen/flank. It may vocalize and/or try to bite. The cat does not allow palpation of the abdomen/flank	3
**Vocalization**	• The cat is quiet, purring when stimulated, or miaows interacting with the observer, but does not growl, groan, or hiss	0
	• The cat purrs spontaneously (without being stimulated or handled by the observer)	1
	• The cat growls, howls, or hisses when handled by the observer (when its body position is changed by the observer)	2
	• The cat growls, howls, hisses spontaneously (without being stimulated or handled by the observer)	3
	**Subscale 2: Psychomotor change (0 – 12)**	
**Posture**	• The cat is in a natural posture with relaxed muscles (it moves normally)	0
	• The cat is in a natural posture but is tense (it moves little or is reluctant to move)	1
	• The cat is sitting or in sternal recumbency with its back arched and head down; or The cat is in dorso-lateral recumbency with its pelvic limbs extended or contracted	
	• The cat frequently alters its body position in an attempt to find a comfortable posture	3
**Comfort**	• The cat is comfortable, awake or asleep, and interacts when stimulated (it interacts with the observer and/or is interested in its surroundings)	0
	• The cat is quiet and slightly receptive when stimulated (it interacts little with the observer and/or is not very interested in its surroundings)	1
	• The cat is quiet and “dissociated from the environment” (even when stimulated it does not interact with the observer and/or has no interest in its surroundings) The cat may be facing the back of the cage	2
	• The cat is uncomfortable, restless (frequently changes its body position), and slightly receptive when stimulated or “dissociated from the environment” the cat may be facing the back of the cage	3
**Activity**	• The cat moves normally (it immediately moves when the cage is opened; outside the cage it moves spontaneously when stimulated or handled)	0
	• The cat moves more than normal (inside the cage it moves continuously from side to side)	1
	• The cat is quieter than normal (it may hesitate to leave the cage and if removed from the cage tends to return, outside the cage it moves a little after stimulation or handling)	2
	• The cat is reluctant to move (it may hesitate to leave the cage and if removed from the cage tends to return, outside the cage it does not move even when stimulated or handled)	3
**Attitude**	Observe and mark the presence of the mental states listed below	
	**A** - **Satisfied:** The cat is alert and interested in its surroundings (explores its surroundings), friendly and interactive with the observer (plays and/or responds to stimuli) *The cat may initially interact with the observer through games to distract it from the pain. Carefully observe to distinguish between distraction and satisfaction games	A
	**B** - **Uninterested:** The cat does not interact with the observer (not interested by toys or plays a little; does not respond to calls or strokes from the observer) * In cats which don’t like to play, evaluate interaction with the observer by its response to calls and strokes	B
	**C** - **Indifferent:** The cat is not interested in its surroundings (it is not curious; it does not explore its surroundings) * The cat can initially be afraid to explore its surroundings. The observer needs to handle the cat and encourage it to move itself (take it out of the cage and/or change its body position)	C
	**D** - **Anxious:** The cat is frightened (it tries to hide or escape) or nervous (demonstrating impatience and growling, howling, or hissing when stroked and/or handled)	D
	**E** - **Aggressive:** The cat is aggressive (tries to bite or scratch when stroked or handled)	E
	• Presence of the mental state A	0
	• Presence of one of the mental states B, C, D, or E	1
	• Presence of two of the mental states B, C, D, or E	2
	• Presence of three or all of the mental states B, C, D, or E	3
	**Subscale 3: Physiological variables (0 – 6)**	
**Arterial blood pressure**	• 0% to 15% above pre-surgery value	0
	• 16% to 29% above pre-surgery value	1
	• 30% to 45% above pre-surgery value	2
	• > 45% above pre-surgery value	3
**Appetite**	• The cat is eating normally	0
	• The cat is eating more than normal	1
	• The cat is eating less than normal	2
	• The cat is not interested in food	3
	**Directions for using the scale**	
Initially observe the cat’s behavior without opening the cage. Observe whether it is resting or active; interested or uninterested in its surroundings; quiet or vocal. Check for the presence of specific behaviors (see “Miscellaneous behaviors” above).		
Open the cage and observe whether the cat quickly moves out or hesitates to leave the cage. Approach the cat and evaluate its reaction: friendly, aggressive, frightened, indifferent, or vocal. Touch the cat and interact with it, check whether it is receptive (if it likes to be stroked and/or is interested in playing). If the cat hesitates to leave the cage, encourage it to move through stimuli (call it by name and stroke it) and handling (change its body position and/or take it out of the cage). Observe when outside the cage, if the cat moves spontaneously, in a reserved manner, or is reluctant to move. Offer it palatable food and observe its response.*		
Finally, place the cat in lateral or sternal recumbency and measure its arterial blood pressure. Evaluate the cat’s reaction when the abdomen/flank is initially touched (slide your fingers over the area) and the in sequence gently pressed (apply direct pressure over the area). Wait for a time, and do the same procedure to assess the cat’s reaction to palpation of surgical wound.		
*To evaluate appetite during the immediate postoperative period, initially offer a small quantity of palatable food immediately after recovery from anaesthetic. At this moment most cats eat normally independent of the presence or absence of pain. Wait a short while, offer food again, and observe the cat’s reaction.		

### Construct validity by factor analysis

Exploratory factor analysis supported the multidimensionality previously observed in the original Portuguese scale but revealed a three-factor solution with eigenvalues of 3.07, 3.04 and 1.20. Factor 1 labeled ‘pain expression’ explained 30.7% of the variance included the *miscellaneous behaviors*, *reaction to palpation of surgical wound*, *reaction to palpation of abdomen/flank* and *vocalization*. Factor 2 or ‘psychomotor changes’ accounted for 30.4% of the variance, and included *posture*, *comfort*, *activity* and *attitude*. The third factor named ‘physiological variables’ included *arterial blood pressure* and *appetite* and contributed to 12.0% of the total variance. The score for ‘pain expression’ and ‘psychomotor change’ subscales ranged from 0 to 12 points; and for ‘physiological variables’ subscale ranged from 0 to 6 points.

### Phase 1: Validity and reliability testing based on video analysis

#### Criterion validity by comparison with a gold standard

At all time points the agreement between blinded observers and the ‘gold-standard’ observer as evaluated by weighted kappa coefficient, was very good for all scale items. When T2 was independently assessed agreement ranged from moderate to very good. The items *activity*, *attitude* and *comfort* showed the lowest agreement (Table 2).

**Table 2:** Agreement between blinded observers and ‘gold standard’ for each scale item – video analysis

**Items**	**Blinded observers**				
	**Anesthesiologist 1**	**Anesthesiologist 2**	**Anesthesia technician 1**	**Anesthesia technician 2**	**PhD student**
Posture	**0.96** (0.94 - 0.97)	**0.96** (0.95 - 0.97)	**0.98** (0.97 - 0.99)	**0.97** (0.96 - 0.98)	**0.94** (0.91 - 0.96)
	***0.90****(0.79 - 0.95)*	***0.90****(0.81 - 0.95)*	***0.89****(0.79 - 0.95)*	***1.00***	***0.95****(0.89 - 0.97)*
Comfort	**0.95** (0.93 - 0.97)	**0.98** (0.97 - 0.98)	**0.94** (0.91 - 0.96)	**0.93** (0.90 - 0.95)	**0.95** (0.93 - 0.97)
	***0.85****(0.72 - 0.93)*	***0.96****(0.92 - 0.98)*	***0.85****(0.70 - 0.93)*	***0.70****(0.47 - 0.85)*	***0.55****(0.24 - 0.75)*
Activity	**0.93** (0.90 - 0.95)	**0.91** (0.88 - 0.94)	**0.91** (0.87 - 0.94)	**0.88** (0.83 - 0.92)	**0.92** (0.89 - 0.95)
	***0.86****(0.74 - 0.93)*	***0.66****(0.40 - 0.82)*	***0.60****(0.31 - 0.79)*	***0.69****(0.45 - 0.84)*	***0.73****(0.50 - 0.87)*
Attitude	**0.93** (0.89 - 0.95)	**0.95** (0.93 - 0.97)	**0.94** (0.92 - 0.96)	**0.90** (0.86 - 0.93)	**0.96** (0.95 - 0.97)
	***0.70****(0.37 - 0.86)*	***0.76****(0.57 - 0.88)*	***0.75****(0.55 - 0.87)*	***0.77****(0.52 - 0.89)*	***0.96****(0.92 - 0.98)*
Miscellaneous behaviors	**0.99** (0.98 - 0.99)	**0.97** (0.95 - 0.98)	**0.96** (0.94 - 0.97)	**0.96** (0.94 - 0.97)	**0.95** (0.93 - 0.96)
	***0.82****(0.67 - 0.91)*	***0.86****(0.73 - 0.93)*	***0.84****(0.70 - 0.92)*	***0.88****(0.77 - 0.94)*	***0.65****(0.38 - 0.81)*
Reaction to palpation of the surgical wound	**0.97** (0.95 - 0.98)	**0.96** (0.94 - 0.97)	**0.98** (0.97 - 0.99)	**0.95** (0.92 - 0.96)	**0.95** (0.92 - 0.96)
	***0.89****(0.77 - 0.95)*	***0.93****(0.86 - 0.97)*	***0.95****(0.90 - 0.98)*	***0.84****(0.70 - 0.92)*	***0.90****(0.81 - 0.96)*
Reaction to the palpation of the abdomen/flank	**0.93** (0.90 - 0.95)	**0.93** (0.90 - 0.95)	**0.95** (0.94 - 0.97)	**0.95** (0.93 - 0.97)	**0.94** (0.92 - 0.96)
	***0.88****(0.76 - 0.94)*	***0.87****(0.74 - 0.94)*	***0.87****(0.74 - 0.94)*	***0.84****(0.70 - 0.92)*	***0.87****(0.74 - 0.94)*
Appetite	**0.99** (0.98 - 0.99)	**0.92** (0.89 - 0.94)	**0.94** (0.92 - 0.96)	**0.96** (0.95 - 0.97)	**0.95** (0.93 - 0.97)
	***0.98****(0.96 - 0.99)*	***0.89****(0.79 - 0.95)*	***0.94****(0.87 - 0.97)*	***0.94****(0.88 - 0.97)*	***0.94****(0.88 - 0.97)*
Vocalization	**0.95** (0.93 - 0.96)	**0.97** (0.96 - 0.98)	**0.90** (0.86 - 0.93)	**0.97** (0.96 - 0.98)	**0.90** (0.86 - 0.93)
	***0.98****(0.95 - 0.99)*	***0.95****(0.89 - 0.97)*	***0.83****(0.63 - 0.92)*	***0.97****(0.95 - 0.99)*	***0.90****(0.79 - 0.95)*

Agreement between blinded observers and ‘gold standard’, assessed by weighted kappa coefficient (95% CI), for each scale item, at all time points (preoperative, postoperative before and after analgesia, and 24 hours after the end of surgery) and for *T2 (postoperative before analgesia)* independently.

Interpretation: 0.81 – 1.0 very good; 0.61 – 0.80 good; 0.41 – 0.6 moderate; 0.21 – 0.4 fair; < 0.2 poor.

#### Construct validity by hypotheses testing

Since factor analysis confirmed the multidimensionality of the English version of the scale, the construct validity was determined for both total and partial or subscale scores. These increased significantly at T2 (after surgery but before postoperative analgesics) when compared to T1 (preoperative). They decreased significantly after cats received postoperative analgesics (T2 vs. T3) and over time from T2 to T4 (Table 3).

**Table 3:** Total and partial pain scores determined by blinded observers and ‘gold standard’ – video analysis

**Evaluation times**	**Pain scores**	**Gold standard**	**Blinded observers**				
			**Anesthesiologist 1**	**Anesthesiologist 2**	**Anesthesia technician 1**	**Anesthesia technician 2**	**PhD student**
T1 Preoperative	Total	0.0 (5.0)	0.0 (5.0)	0.0 (6.0)	0.0 (5.0)	1.0 (4.0)	0.0 (6.0)
	(0–30)						
	Subscale 1	0.0 (0.0)	0.0 (1.0)	0.0 (2.0)	0.0 (1.0)	0.0 (1.0)	0.0 (0.0)
	(0 – 12)						
	Subscale 2	0.0 (5.0)	0.0 (5.0)	0.0 (5.0)	0.0 (5.0)	0.0 (4.0)	0.0 (6.0)
	(0 – 12)						
	Subscale 3	0.0 (0.0)	0.0 (0.0)	0.0 (2.0)	0.0 (0.0)	0.0 (0.0)	0.0 (0.0)
	(0 – 6)						
T2 Postoperative: before analgesia	Total	20.5 (14.0)*	20.0 (15.0)*	22.0 (15.0)*	20.0 (14.0)*	21.0 (15.0)*	20.0 (16.0)*
	(0 – 30)						
	Subscale 1	8.0 (10.0)*	7.0 (10.0)*	8.5 (10.0)*	7.5 (11.0)*	7.5 (10.0)*	7.0 (11.0)*
	(0 – 12)						
	Subscale 2	9.5 (6.0)*	9.0 (6.0)*	10.0 (7.0)*	10.0 (6.0)*	9.0 (7.0)*	10.0 (8.0)*
	(0 – 12)						
	Subscale 3	3.0 (6.0)*	3.0 (6.0)*	3.0 (6.0)*	3.0 (6.0)*	3.0 (6.0)*	3.0 (6.0)*
	(0 – 6)						
T3 Postoperative: after analgesia	Total	0.0 (6.0) †	0.0 (4.0) †	0.0 (4.0) †	1.0 (6.0) †	1.0 (4.0) †	1.0 (5.0) †
	(0 – 30)						
	Subscale 1	0.0 (1.0) †	0.0 (1.0) †	0.0 (3.0) †	0.0 (1.0) †	0.0 (1.0) †	0.0 (2.0) †
	(0 – 12)						
	Subscale 2	0.0 (5.0) †	0.0 (3.0) †	0.0 (3.0) †	0.0 (5.0) †	0.0 (4.0) †	0.0 (5.0) †
	(0 – 12)						
	Subscale 3	0.0 (2.0) †	0.0 (2.0) †	0.0 (2.0) †	0.0 (4.0) †	0.0 (1.0) †	0.0 (4.0) †
	(0 – 6)						
T4 Postoperative:	Total	4.0 (14.0) †	3.0 (11.0) †	3.0 (14.0) †	4.0 (14.0) †	4.0 (13.0) †	3.0 (11.0) †
24 hours after end of surgery	(0 – 30)						
	Subscale 1	2.0 (6.0) †	2.0 (7.0) †	2.0 (7.0) †	2.0 (6.0) †	3.0 (8.0) †	2.0 (6.0) †
	(0 – 12)						
	Subscale 2	0.0 (6.0) †	0.0 (5.0) †	0.0 (5.0) †	0.0 (6.0) †	0.0 (6.0) †	0.0 (6.0) †
	(0 – 12)						
	Subscale 3	0.0 (2.0) †	0.0 (2.0) †	0.0 (3.0) †	0.0 (2.0) †	0.0 (2.0) †	0.0 (2.0) †
	(0 – 6)						

Median and range of the total and partial pain scores determined by blinded observers and ‘gold standard’ based on video analysis recorded at 4 time points during the perioperative period in cats undergoing ovariohysterectomy.

* Pain scores in T2 significantly higher than T1 (p < 0.001).

† Pain scores in T3 and T4 significantly lower than T2 (p < 0.001).

Subscale 1 ‘Pain expression’ - *miscellaneous behaviors, reaction to palpation of the surgical wound, reaction to palpation of the abdomen/flank and vocalization*.

Subscale 2 ‘Psychomotor change’ - *posture, comfort, activity and attitude.*

Subscale 3 ‘Physiological variables’ - *arterial blood pressure and appetite.*

#### Responsiveness

The absolute and percent decrease in pain scores (mean ± standard deviation) in response to rescue analgesia and over time were 19 ± 4 (95% ± 6.4), and 16 ± 4 (81.6% ± 16.7), respectively. Relative to the maximum score of the UNESP-Botucatu-MCPS, the pain scores changed 64.5% ± 15.5, 64.1% ± 14.1, and 54.8% ± 15.5, after surgery, administration of analgesics and over time, respectively.

#### Inter-rater reliability

At all time points, the agreement among blinded observers, assessed by ICC was very good for all scale items, the total and subscale scores. When T2 was independently assessed, the agreement ranged from moderate to very good. Items labeled *activity*, *attitude* and *comfort* in the scale showed the lowest agreement (Table 4).

**Table 4:** Agreement among blinded observers for each item and total and partial pain scores – video analysis

**Items**	**Agreement among blinded observers**	
	**All assessment times**	**T2 separately**
Posture	**0.94** (0.92 - 0.98)	**0.86** (0.78 - 0.92)
Comfort	**0.92** (0.89 - 0.94)	**0.61** (0.46 - 0.76)
Activity	**0.86** (0.82 - 0.89)	**0.57** (0.41 - 0.73)
Attitude	**0.91** (0.88 - 0.93)	**0.66** (0.52 - 0.79)
Miscellaneous behaviors	**0.94** (0.92 - 0.95)	**0.73** (0.60 - 0.84)
Reaction to palpation of surgical wound	**0.92** (0.90 - 0.94)	**0.81** (0.71 - 0.89)
Reaction to palpation of abdomen/flank	**0.89** (0.87 - 0.92)	**0.78** (0.66 - 0.88)
Appetite	**0.93** (0.91 - 0.95)	**0.92** (0.87 - 0.96)
Vocalization	**0.89** (0.86 - 0.91)	**0.88** (0.81 - 0.93)
**Total Score**	**0.98** (0.98 - 0.99)	**0.92** (0.87 - 0.96)
**Partial Score Subscale 1** ‘Pain Expression’	**0.97** (0.96 - 0.98)	**0.92** (0.87 - 0.96)
**Partial Score Subscale 2** ‘Psychomotor change’	**0.96** (0.95 - 0.97)	**0.81** (0.71 - 0.89)

Agreement among blinded observers, assessed by intra-class correlation coefficient (95% CI), for each scale item and total and partial scores from subscales 1 and 2, at all time points (preoperative, postoperative before and after analgesia, and 24 hours after the end of surgery), and for T2 (postoperative before analgesia) independently.

Interpretation: 0.81 – 1.0 very good; 0.61 – 0.80 good; 0.41 – 0.6 moderate; 0.21 – 0.4 fair; < 0.2 poor.

#### Intra-rater reliability

The intra-rater reliability determined by ICC was very good for all scale items. When T2 was independently assessed, intra-rater reliability was moderate to very good. *Appetite* and *attitude* showed the highest and lowest agreement, respectively (Table 5).

**Table 5:** Intra-rater reliability for each scale item – video analysis

**Items**	**Blinded observers**				
	**Anesthesiologist 1**	**Anesthesiologist** **2**	**Anesthesia technician 1**	**Anesthesia technician 2**	**PhD student**
Posture	**0.98** (0.97 - 0.99)	**0.98** (0.97 - 0.99)	**0.97** (0.96 - 0.98)	**0.97** (0.96 - 0.98)	**0.98** (0.97 - 0.98)
	***0.95****(0.90 - 0.97)*	***0.86****(0.75 - 0.93)*	***0.76****(0.57 - 0.88)*	***0.95****(0.89 - 0.97)*	***0.89****(0.79 - 0.95)*
Comfort	**0.98** (0.97 - 0.99)	**0.98** (0.98 - 0.99)	**0.97** (0.95 - 0.98)	**0.96** (0.95 - 0.97)	**0.99** (0.98 - 0.99)
	***0.93****(0.87 - 0.97)*	***0.89****(0.77 - 0.94)*	***0.89****(0.77 - 0.95)*	***0.88****(0.77 - 0.94)*	***0.90****(0.80 - 0.95)*
Activity	**0.92** (0.89 - 0.94)	**0.94** (0.91 - 0.95)	**0.93** (0.91 - 0.95)	**0.91** (0.87 - 0.94)	**0.92** (0.88 - 0.94)
	***0.66****(0.41 - 0.82)*	***0.92****(0.84 - 0.96)*	***0.80****(0.62 - 0.90)*	***0.83****(0.68 - 0.92)*	***0.72****(0.50 - 0.86)*
Attitude	**0.94** (0.92 - 0.96)	**0.96** (0.95 - 0.97)	**0.96** (0.94 - 0.97)	**0.96** (0.94 - 0.97)	**0.98** (0.97 - 0.98)
	***0.79****(0.60 - 0.90)*	***0.57****(0.27 - 0.77)*	***0.85****(0.70 - 0.93)*	***0.85****(0.71 - 0.93)*	***0.90****(0.80 - 0.95)*
Miscellaneous behaviors	**0.98** (0.98 - 0.99)	**0.96** (0.94 - 0.97)	**0.96** (0.95 - 0.97)	**0.93** (0.90 - 0.95)	**0.96** (0.94 - 0.97)
	***0.87****(0.75 - 0.94)*	***0.83****(0.67 - 0.92)*	***0.74****(0.53 - 0.87)*	***0.80****(0.62 - 0.90)*	***0.71****(0.46 - 0.85)*
Reaction to palpation of surgical wound	**0.96** (0.95 - 0.97)	**0.94** (0.91 - 0.97)	**0.99** (0.98 - 0.99)	**0.96** (0.95 - 0.97)	**0.96** (0.95 - 0.97)
	***0.92****(0.82 - 0.96)*	***0.95****(0.89 - 0.97)*	***0.97****(0.95 - 0.99)*	**0.94** (0.87 - 0.97)	***0.90****(0.81 - 0.95)*
Reaction to palpation of abdomen/flank	**0.94** (0.91 - 0.95)	**0.95** (0.93 - 0.97)	**0.99** (0.98 - 0.99)	**0.95** (0.94 - 0.97)	**0.95** (0.93 - 0.96)
	***0.88****(0.77 - 0.94)*	***0.95****(0.90 - 0.97)*	***0.95****(0.90 - 0.98)*	***0.85****(0.71 - 0.93)*	***0.95****(0.89 - 0.97)*
Appetite	**0.96** (0.95 - 0.97)	**0.94** (0.92 - 0.96)	**0.95** (0.93 - 0.96)	**0.97** (0.96 - 0.98)	**0.99** (0.98 - 0.99)
	***0.94****(0.87 - 0.97)*	***0.92****(0.84 - 0.96)*	***0.94****(0.88 - 0.97)*	***0.96****(0.92 - 0.98)*	***0.98****(0.96 - 0.99)*
Vocalization	**0.96** (0.95 - 0.97)	**0.95** (0.93 - 0.96)	**0.90** (0.86 - 0.93)	**0.93** (0.90 - 0.95)	**0.92** (0.89 - 0.95)
	***0.97****(0.95 - 0.99)*	***0.91****(0.82 - 0.95)*	***0.82****(0.66 - 0.91)*	***0.92****(0.85 - 0.96)*	***0.91****(0.83 - 0.96)*

Intra-rater reliability assessed by intra-class correlation coefficient (95% CI) for each scale item, at all time points (preoperative, postoperative before and after analgesia, and 24 hours after the end of surgery) and for *T2 (postoperative before analgesia)* independently.

Interpretation: 0.81 – 1.0 very good; 0.61 – 0.80 good; 0.41 – 0.6 moderate; 0.21 – 0.4 fair; < 0.2 poor.

#### Cut-off point for rescue analgesia

**Figure 1:**
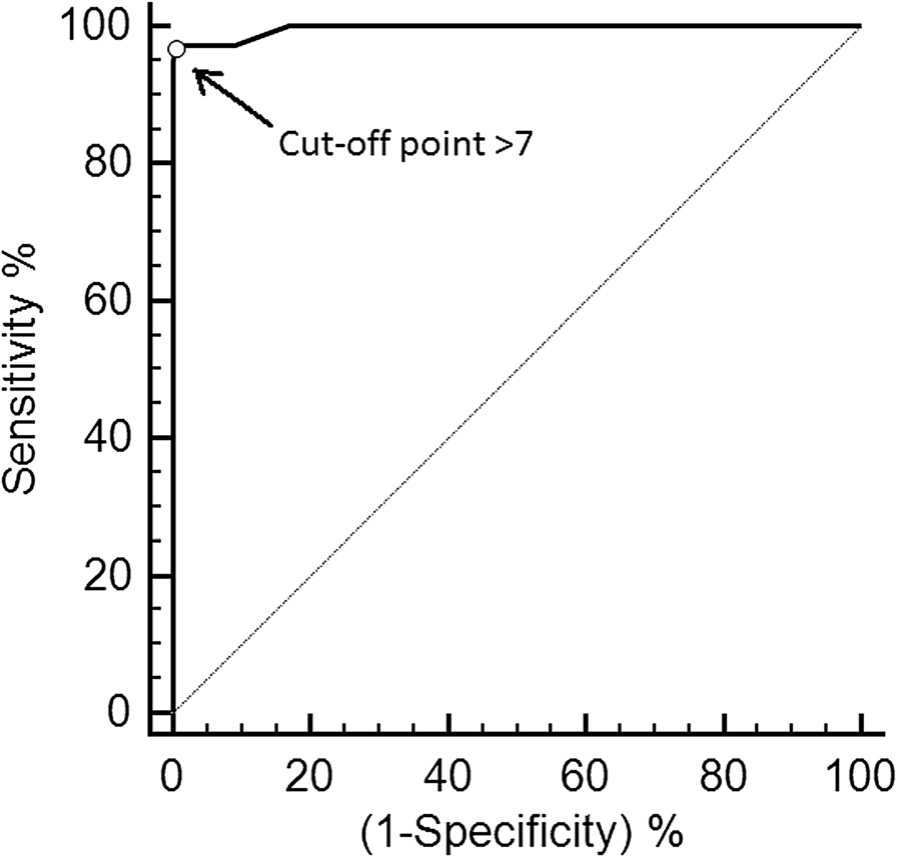
**ROC curve and the optimal cut-off point > 7 for rescue analgesia.** Receiver Operating Characteristic ROC curve showing the optimal cut-off point > 7 for rescue analgesia (0 – 30 points), with 96.5% of sensitivity, 99.5% of specificity and AUC of 0.996, based on analysis of videos recorded at 4 time points during the perioperative period in cats undergoing ovariohysterectomy.

**Figure 2:**
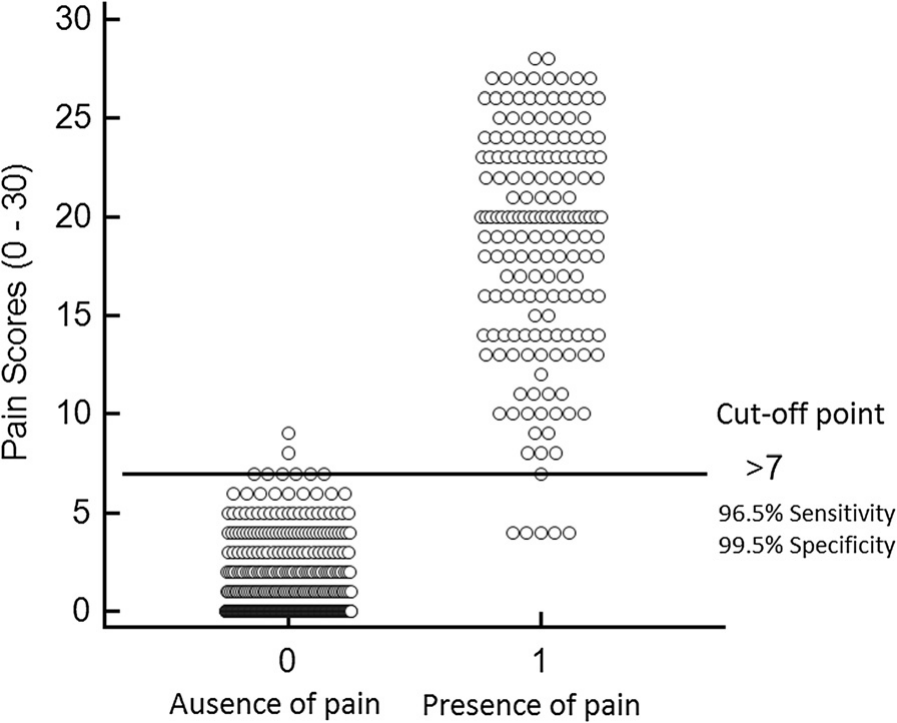
**Diagram illustrating the optimal cut-off point for rescue analgesia.** Diagram illustrating the optimal cut-off point for rescue analgesia identified from the analysis of the ROC curve, based on analysis of videos recorded at 4 time points during the perioperative period in cats undergoing ovariohysterectomy.

From the analysis of the ROC curve, different cut-off points were suggested, highlighting the point represented by the greatest value of the sensitivity and specificity, simultaneously. The optimal cut-off point identified was > 7 (scale range 0 – 30 points), with a sensitivity of 96.5% (95% CI: 92.6 – 98.7%), and specificity of 99.5% (95% CI: 98.3 – 99.9%). The high AUC = 0.996 (95% CI: 0.987 – 0.999; p < 0.001) indicated that the instrument has excellent discriminatory ability (Figures 1 and 2).

A cut-off point above which interventional analgesics are to be recommended was also calculated for subscales that substantially contributed for the total variance of the scale, as subscales 1 and 2. For subscale 1 ‘pain expression’ the optimal cut-off point was > 2 (scale range 0 – 12 points), with 94.8% of sensitivity (95% CI: 90.4 – 97.6%), 89.9% of specificity (95% CI: 86.6 – 92.6%), and AUC = 0.984 (95% CI: 0.970 – 0.992; p < 0.001). The cut-off point for subscale 2 ‘psychomotor change’ was > 3 (scale range 0 – 12 points), with 93.1% of sensitivity (95% CI: 88.3 – 96.4%), 93.9% of specificity (95% CI: 91.2 – 96.0%), and AUC = 0,969 (95% CI: 0.952 – 0.981; p < 0.001).

### Phase 2: Validity and reliability testing based on clinical application of the scale in an English speaking country

#### Internal consistency

Cronbach’s alpha coefficient for the total score was 0.84, which indicated excellent internal consistency. The internal consistency of the subscales 1 ‘pain expression’ and 2 ‘psychomotor change’ were also excellent at 0.86 and 0.87, respectively. Subscale 3 ‘physiological variables’ showed unacceptable internal consistency with a value of 0.28.

#### Construct validity by known-group discrimination

**Figure 3:**
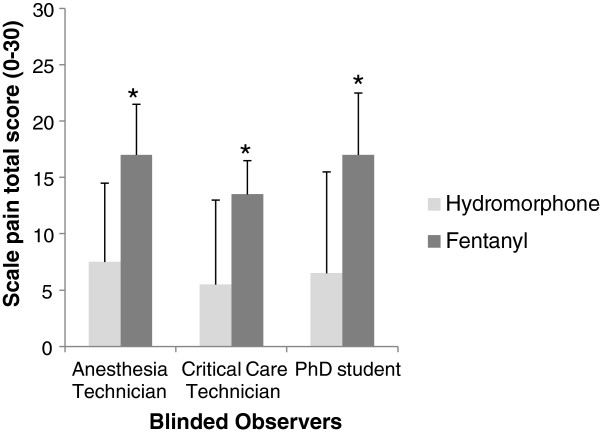
**Construct validity by known-groups discrimination: perioperative hydromorphone or preoperative fentanyl.** Median and semi-range (range divided by 2) of the total pain score one hour post anesthesia in cats undergoing ovariohysterectomy and treated with either perioperative hydromorphone (n = 16) or preoperative fentanyl (n = 12), during the clinical application of the English version of the UNESP-Botucatu-MCPS in an English-speaking country (Veterinary Teaching Hospital - Colorado State University, USA). * Indicates statistical difference between groups (p < 0.001) by Mann–Whitney test.

**Figure 4:**
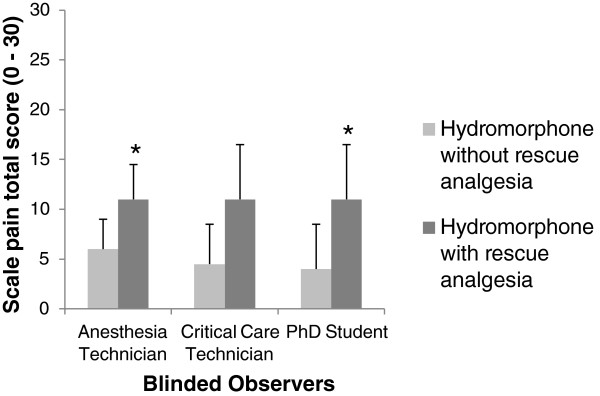
**Construct validity by known-groups discrimination: perioperative hydromorphone that required or did not require rescue analgesia.** Median and semi-range (range divided by 2) of the total pain score one hour post anesthesia in cats undergoing ovariohysterectomy and treated with perioperative hydromorphone, that required (n = 5) or did not require (n = 11) rescue analgesia, during the clinical application of the English version of the UNESP-Botucatu-MCPS in an English-speaking country (Veterinary Teaching Hospital - Colorado State University, USA). * Indicates statistical difference between groups (p < 0.01) by Mann–Whitney test.

At the 1 hour post-operative time point, the total and partial subscale scores were able to distinguish cats receiving hydromorphone from those receiving only fentanyl (p < 0.05), with one exception noted for subscale 1 for the critical care technician (p = 0.07). When assessing just the hydromorphone group, both the total score and that from subscale 1 discriminated cats that required rescue analgesia from cats that did not (p < 0.01), again with the exception for total score for the critical care technician (p > 0.05) (Figures 3 and 4).

#### Concurrent validity (criterion validation)

In considering all assessment times (pre and postoperative) a high correlation was noted between pain scores determined by the English version of the UNESP-Botucatu-MCPS and the IVAS scores for all blinded observers: anesthesia technician (r = 0.87; p < 0.000), critical care technician (r = 0.78; p < 0.000) or anesthesiology PhD student (r = 0.92; p < 0.000).

#### Inter-rater reliability

The agreement among blinded observers ranged from good to very good for all scale items: *posture* 0.82 (CI: 0.76 – 0.86); *comfort* 0.83 (CI: 0.78 – 0.87); *activity* 0.81 (CI: 0.75 – 0.85); *attitude* 0.77 (CI: 0.70 – 0.82); *miscellaneous behaviors* 0.77 (CI: 0.70 – 0.83); *reaction to palpation of surgical wound* 0.86 (CI: 0.80 – 0.90); *reaction to palpation of abdomen/ flank* 0.85 (CI: 0.77 – 0.87), *appetite* 0.97 (CI: 0.96 – 0.98) and *vocalization* 0.83 (CI: 0.78 – 0.87).

## Discussion

In this study, the original UNESP-Botucatu-MCPS in Brazilian Portuguese was first translated into English as described. Then the validity and reliability of the English version were evaluated first through assessment of perioperative video recordings at different time points and then by application of scale in a clinical setting in an English-speaking country. The results confirmed the multidimensional structure of the scale, and attested its validity and reliability when used by anesthesiologists or anesthesia technicians for assessing acute pain in cats undergoing OHE. Furthermore, we were able to determine a value above which rescue analgesic administration is recommended. Similar to original scale in Brazilian Portuguese [16-18], the validity and reliability of the English version of the UNESP-Botucatu-MCPS were excellent, supporting that the translation and cultural adaptation were appropriate.

The assessment of the internal structure of a scale by factor analysis is a method used to establish the construct validity of a tool [19,20]. The factor structure of the English version of the UNESP-Botucatu-MCPS showed some differences when compared to the 4-factor solution observed in the original scale in Portuguese [16]. Hence, items were reorganized and placed in different subscales in the English version. Factor analysis identified three dimensions or subscales in the English version that were named: ‘pain expression’, ‘psychomotor change’, and ‘physiological variables’. The items that composed the subscales ‘physiological variables’ and ‘psychomotor change’ were the same of the original scale, except for the item *miscellaneous behaviors* that in the English version was included in the subscale ‘pain expression’. The dimension ‘pain expression’, gathered the subscale ‘protection of wound area’ and ‘vocal expression of pain’ of the original scale, plus the item *miscellaneous behaviors*. The 3-factor structure observed in the English version of the UNESP-Botucatu-MCPS is more appropriate than the 4-factors structure of original scale in Portuguese.

While the internal consistency of the total score and partial scores from subscales ‘pain expression’ and ‘psychomotor change’ were excellent, the subscale titled ‘physiological variables’ showed an unacceptable internal consistency. This was different from the results observed in the original scale in which internal consistency for this subscale was very good [16]. A potential explanation is the difference in *arterial blood pressure* relative to different methodology in the two studies. In the current study, low variability was noted in arterial *blood pressure* measurements as both treatment groups received opioids. In the original study the presence of a control group (absence of analgesics) likely produced greater variability in blood pressure readings when compared to cats receiving analgesics [21].

In the current study however the subscale titled ‘physiological variables’ was able to distinguish between cats treated with hydromorphone or fentanyl at one hour after extubation. We therefore believe that the discriminative ability of this subscale in the immediate postoperative period justifies its inclusion. However, given its poor internal consistency it should only be used in association with subscales 1 and 2. Alternatively given this subscale contributed to only 12% of the total variance, it could be omitted without compromising the global pain assessment, especially if technical difficulty is encountered when assessing physiological parameters.

The typical methodology for examining criterion validity is concurrent validity which correlates the new scale against another instrument, ideally, a ‘gold standard’ [4]. This approach has been used to validate pain scales in veterinary [7,15], and human medicine [22,23]. However, to the authors’ knowledge there is no ‘gold standard’ tool to assess acute pain in cats, since scales that are usually used in this species, like SDS, IVAS and NRS have not been tested for validity and reliability. Taking into account that the IVAS has superior measurement property when compared to the other scales above cited, it was decided to correlate the total score of the English version of the UNESP-Botucatu-MCPS with the scores determined by IVAS. In veterinary medicine where zero score is arbitrary, because complete absence to pain based on behavior evaluation cannot be assumed, the IVAS provides interval level measurements [7]. In order to avoid the possibility of the global pain assessment obtained from use of the IVAS influencing the MCPS scores, the blinded observers were instructed to complete the MCPS first, and then the IVAS. The high correlation observed between these scales helped to establish concurrent validity.

However the approach aforementioned may be disputable. Because of that, an alternative method to assess criterion validity, similar to that was described by Gauvain-Piquard et al. to validate a pain scale for young children with cancer was also used [24]. This method is based on the agreement between pain scores recorded by blinded observers, and the ‘gold standard’ observer, who in this study was the investigator that developed the scale and who also has advanced training and significant experience in feline pain assessment. Except for *comfort*, and *activity* that showed only moderate agreement, the other items had good to very good agreement between blinded observers and ‘gold standard’ evaluator. This result was similar to that observed in the original scale in Portuguese, where only *activity* showed moderate agreement [17], and confirmed the criterion validity of the English version of the UNESP-Botucatu-MCPS.

Although the content validity may be established based on the opinion of a committee of experts in the target field, construct validation is an ongoing process that can be evaluated in numerous ways [4,19]. In the first phase of this study (video analysis) the construct validity was tested based on the hypotheses that time and intervention (surgery, administration of analgesics) would change the pain scores. The intervention approach has been extensively used to validate pain scales in human pediatrics [23,25,26], whereas change in scores over time is described for use in veterinary medicine [7,15]. Similar to the results of the validation of the original scale in Portuguese [17], the total and subscales pain scores of the English version increased in response to surgery, and decreased after postoperative analgesics and with time, together supporting construct validity.

The construct validity was also assessed using the known-group method, a kind of validity which determines whether the instrument is able to detect differences between groups [19,27]. This technique has been previously used to validate tools to measure chronic pain in dogs [28]. In this study, this methodology was applied by using the English version of the scale to evaluate the analgesic efficacy of perioperative hydromorphone or preoperative fentanyl in cats undergoing OHE. The total and partial subscales scores were able to distinguish between different analgesic treatments in the immediate postoperative period. Both the total score and score from subscale 1 also distinguished between cats requiring additional analgesia in the hydromorphone group. This good discriminatory ability of the English version is consistent with the results obtained using the original scale in Portuguese where cats treated with analgesics and those receiving only placebo could be distinguished [21].

Responsiveness or sensitivity to change reflects the ability of an instrument to detect significant changes in pain scores in the expected direction [29]. The change in pain scores either in response to analgesic administration or over time, have been used to assess the responsiveness of the instruments to measure pain in dogs with chronic osteoarthritis [30,31], or acute pain [7,15]. In this study, the responsiveness of the English version of the UNESP-Botucatu-MCPS was supported by the significant change in pain scores in response to surgery, administration of postoperative analgesics and over time.

Pain scores decreased an average of 95% and 81%, following analgesic treatment and over time, respectively. The percent of decrease in pain scores after postoperative analgesia (T2 vs T3) was greater than over time (T2 vsT4). This is likely because T3 was in close proximity to administration of multiple analgesic medications (morphine, ketoprofen and dipyrone) whereas at T4 it is likely only the longer acting NSAID, ketoprofen was effective. In humans, the percentage of reduction in pain scores above of 30% or 55% has been proposed as clinically meaningful [32-34]. However, the UNESP-Botucatu-MCPS has an ordinal level of measurement and so calculation of the percent of change is not generally recommended. On the other hand, this technique has been used to assess the responsiveness of the NRS, also an ordinal scale [32,33]. The explanation for this procedure is that like the NRS, the UNESP-Botucatu-MCPS provides the global magnitude of pain assessment, different from a SDS that just classifies pain intensity [35].

Additionally, assuming that in composite scales the pain intensity is reflected in the total pain score (sum of the scores for each item) we considered the percentage of change in pain scores, after surgery, postoperative analgesia and time, in relation to the maximum score of the scale (30 points). The pain scores changed an average of 64% after surgery and interventional analgesia, and about 55% with time postoperatively, demonstrating that the scale has the ability to respond in an expected direction. Another point that should be clarified is that the sensitivity to change or responsiveness is not only a characteristic inherent of an instrument, but it is also related to the effects of an intervention [4]. Therefore, the high percent of change in pain scores observed in the current study also reflected the power of the intervention that was used, like a surgery that produces moderate pain (OHE), and postoperative analgesia with a multimodal approach: an opioide (morphine), a non-steroidal anti-inflammatory drug (ketoprofen), and central analgesic (dipyrone).

In the study reported here the inter-rater reliability ranged from moderate to very good, with the lowest agreement noted when T2 was independently assessed. This likely occurred in part because the cats were in pain during this time, and the blinded observers selected a numerical score (1, 2 or 3) based on the identification of pain behaviors, and not simply the observation of the absence of pain (score 0). As observed in the original scale, the items *comfort*, *activity* and *attitude* showed the lowest agreement, while the *miscellaneous behaviors* showed better agreement in the English version when compared to the original in Portuguese [18]. In relation to the intra-rater reliability, good to very good agreement values were found for all scale items, as was observed in the original scale [18]. Thus, the English version of the UNESP-Botucatu-MCPS showed adequate reliability when used by the anesthesiologists and anesthesia technicians. We restricted the validation of the scale to individuals with anesthesia training, because in the second phase of the study (clinical application) the pain scores recorded by a critical care technician showed variability when compared to blinded observers with training in anesthesia (technician and PhD student). The critical care technician would consistently underestimate the pain scores, likely because he was not able to identify the specific pain behaviors described in the scale.

The favorable performance of the scale in relation to reproducibility and stability with anesthetist evaluators is likely a result of the detailed description of pain behaviors they are able to identify. This in turn is likely to reduce subjectivity during assessment. Unlike human beings where self-reporting is the ‘gold standard’ for pain assessment [36], in animals the recognition and interpretation of behavioral changes by an observer are used [37]. This emphasizes the importance to examine both inter- and intra- reliability of an instrument for assessing pain in cats. Scales that are considered extremely subjective, like VAS, NRS and SDS showed inconsistent results among different observers when used to assess acute pain in dogs [38].

The availability of a criterion for rescue analgesia is a valuable tool in assisting the observer making decisions about analgesic therapy. Together with pain scores, this may also provide an important measure of the efficacy of analgesic therapy [39]. The optimal analgesic intervention score has been identified using discriminant analysis statistics for the short-form of the Glasgow composite pain scale, a validated instrument to assess acute pain in dogs [7,40,41]. In this study as with the original, the analysis of ROC curve was the strategy selected to define the cut-off point for rescue analgesia [18]. This technique which is used to validate pain scale in human pediatric patients [42] allows determination of the ability of a test to discriminate groups, establish an optimal cut point and compare the performance of tests [43].

Using the criterion of balanced sensitivity and specificity, the best cut-off point identified was > 7, which means that the use of additional analgesia is recommended in scores ≥ 8 (0 – 30 points). This represents 26.6% in relation to the maximum total score of the scale, and is in accordance to the results of the original scale in Portuguese [18], and close to the empirical value of 33% adopted for rescue analgesia before validation of the scale [21].

Further work is required to perform the validation of the English version of the UNESP-Botucatu-MCPS in a clinical setting. However, tools that measure pain from a multidimensional perspective often include many items, and hence take a long time to complete. This maybe a limitation to incorporating this scale in a busy clinical practice, but should be weighed against the usefulness of the information it provides. Some alternatives might be the development of a short form of the scale. Another would be to use only the partial score of the subscale 1 ‘Pain expression’ or 2 ‘Psychomotor change’ for global pain assessment, as these subscales retained a considerable amount of variance, and showed the same excellent properties as the scale total score. The optimal point of these subscales for intervention analgesia was also identified with subscale 1 showing better discriminative ability in clinical study in an English-speaking country.

## Conclusions

In summary, the results of the current study provide evidence that the English version of the UNESP-Botucatu-MCPS is a valid, reliable, responsive scale for assessing acute pain in cats undergoing OHE, when used by anesthesiologists and anesthesia technicians. Additionally through this validation process a numerical criterion for provision of additional (rescue) analgesic therapy has been defined. We hope this will assist the observer using this scale in making appropriate clinical decisions related to analgesic therapy. Standardized instruments of pain assessment, validated in different languages/cultures provide information that can be compared across different studies.

## Methods

The methodology used for the translation, cultural adaptation and validation of the English version of the UNESP-Botucatu-MCPS (also referred to as the ‘instrument’) followed procedures that have been proposed by reputable experts in the field of validation of health measurement instruments [4,19] and are in accordance with international guidelines for cross-cultural validation [3-5]. The scale was first translated, then back-translated and the semantic equivalence verified. In sequence, the validity and reliability of the instrument were tested by evaluators first scoring pain in cats whose observed and interactive behaviors were previously videotaped (phase one) and then by using the tool to assess pain in cats in the clinical setting in an English-speaking country (phase two). These two different approaches were independent and comparison between them was not addressed as part of this study.

### Translation, back-translation and semantic equivalence

The original instrument was translated from Brazilian Portuguese into English by two independent translators fluent in both languages. Both translated versions were synthesized into one version by a third translator and the synthesized version then back-translated by a 4th individual, blinded to the original scale; this person was fluent in Brazilian Portuguese and English (the target language). The synthesized and the back-translated versions were compared and reviewed by the investigators involved in the initial development of the scale and minor adjustments were made in order to maintain maximal semantic equivalence.

### Content validity - analysis by a committee of experts

Three individuals with expertise in feline pain management (Dr. Polly Taylor, Dr. Sheilah Robertson, and Dr. Duncan Lascelles), who were not involved in the previously mentioned translations, reviewed the content and comprehensibility of the scale and judged the appropriateness of each item of the instrument using the following classification: 1 = relatively valid, 0 = not sure, -1 = relatively irrelevant. The results were evaluated using previously described methodology [44], in which the total score from all experts for each item within the overall scale was divided by the number of experts. Items with a value less than 0.5 were revised or deleted.

### Phase 1: Validity and reliability testing based on video analysis

This portion of the study was approved by the Institutional Animal Research Ethical Committee of the FMVZ-UNESP-Botucatu under the protocol number of 20/2008.

Thirty mixed breed cats (2.8 ± 0.5 kg; 14.1 ± 5.2 months) determined to be healthy based on physical examination and results of laboratory tests underwent surgical ovariohysterectomy (OHE) via a ventral midline approach. All OHE’s were performed by a single experienced surgeon. Observed and interactive behaviors were recorded at 4 time points during the perioperative period: T1 “preoperative” (between 18 and 24 hours prior to surgery), T2 “between 30 min and 1 hour after the end of surgery and prior to administration of additional analgesics”, T3 “approximately four hours after postoperative analgesia” and T4 “approximately 24 hours after the end of the surgery”.

Cats were anesthetized with propofol^a^ IV (8 mg/kg), fentanyl^b^ (0.002 mg/kg) IV and isoflurane^c^ in 100% of oxygen using a non-rebreathing system. Morphine^d^ (0.2 mg/kg) IM, ketoprofen^e^ (2 mg/kg) SC and dipyrone^f^ (25 mg/kg) IV were administered for postoperative analgesia to all cats at the conclusion of the T2 video recordings approximately 1 hour after the end of the surgery. The order videos taken from each cat were randomized to ensure blinding of observers who would later evaluate these recordings so that knowledge of the time point would not influence the results. Additionally, the surgical area and catheter site were clipped before preoperative assessments and a small piece of micropore™ medical tape was placed over the surgical area to avoid visualization of the presence or absence of the surgical wound.

Five observers, two ACVA Diplomates and two anesthesia technicians with English as a first language, and a veterinarian obtaining a PhD in anesthesiology with English as a second language, watched the videos and recorded pain scores using the English version of the UNESP-Botucatu-MCPS. These blinded observers were provided directions (Table 1) but not trained in the use of the UNESP-Botucatu-MCPS.

### Criterion validity by comparison with a gold standard

The criterion validity was assessed based on agreement between pain scores recorded by the aforementioned blinded observers and pain scores determined by the ‘gold standard’ observer. The reference person used as a ‘gold standard’ was the investigator that developed the scale, and who has advanced training and significant experience in feline pain assessment. The agreement between each blinded observer and the ‘gold standard’ was determined by the weighted Kappa coefficient [45]. Altman’s classification 0.81 - 1.00 very good; 0.61 - 0.80 good; 0.41 - 0.6 moderate; 0.21 - 0.4 fair and < 0.2 poor [46] was used to interpret the weighted kappa coefficient and 95% confidence interval (CI), calculated for each item of the scale. This was done for cumulative results from all time points and for T2 independently.

### Construct validity by hypotheses testing

The methodology used to establish construct validity was based on hypotheses testing. The first premise formulated was that if the scale actually measures pain, the pain scores at postoperative time, before analgesia (T2), would be higher than those assessed during the preoperative time (T1). The second one examined the difference in pain scores after surgery but before analgesic therapy (T2) and again after administration of analgesics (T3). It was assumed that analgesics would reduce pain therefore pain scores would be lower after administration of analgesics. The third hypothesis was that acute pain should diminish over time (T2 vs. T4). Pain scores were summarized as median and range and the Wilcoxon signed rank test was used for statistical comparisons.

### Responsiveness or sensitivity to change

Hypotheses testing was also used to assess the responsiveness of the scale. The absolute (i.e. difference between pre and post-treatment) and the percent decrease in pain scores (i.e. difference between pre- and post-treatment, divided by pre-treatment score and then multiplied by 100) [35] after postoperative analgesia (T2 vs. T3) and over time (T2 vs. T4) were determined from all the blinded observers. The percent change in pain scores relative to the maximum total score of the UNESP-Botucatu-MCPS (or 30 points), in response to surgery (T1 vs. T2), the administration of postoperative analgesics (T2 vs. T3) and over time (T2 vs. T4) was also calculated.

### Inter-rater reliability

The agreement among blinded observers was evaluated using the intra-class correlation coefficient (ICC) [47], consisting of a two-way random effect model and absolute agreement method with 95% CI. The results were interpreted using Altman’s classification as previously described [46]. The ICC was calculated for each item of the scale at all time points and for T2 independently.

### Intra-rater reliability

For intra-rater reliability the observers were asked to reanalyze the videos, about one month after the first assessment. The digital format was rearranged into a new random sequence of animals and evaluation times to avoid the influence of the previous assessment. As stated for inter-rater reliability, the ICC was calculated for each scale item at all time points and for T2 independently.

### Cut-off point for rescue analgesia

To identify the minimum score at which an animal should be administered analgesic therapy, blinded observers were asked to identify animals that needed additional analgesics after watching each video. This decision was made by answering the question “according your clinical experience, do you think it is necessary to provide rescue analgesia?”

The cut-off point to discriminate the need for analgesic treatment was determined by the ROC curve. The ROC curve plots true positive rates (sensitivity) against false positive rates (1 – specificity) for a series of cut-off values, and the area under the curve (AUC) indicates the discriminative ability of a test [48]. This area theoretically ranges from 0.5 (no accuracy) to 1.0 (perfect accuracy). Values between 0.50 and 0.70, 0.70 and 0.90 and over 0.90 represent low, moderate and high accuracy respectively [43].

### Phase 2: Validity and reliability based on clinical application of the scale in an English-speaking country

The English version of the UNESP-Botucatu-MCPS was used to measure pain scores in cats undergoing to OHE in a study conducted at the Veterinary Teaching Hospital, Colorado State University, Fort Collins, USA. The blinded observers participating in this phase of the study had English as first language (one anesthesia and one critical care technician) or second language (a veterinarian, completing her PhD in veterinary anesthesiology whose native language is Thai). Observers were provided directions (Table 1) but no other training in use of the scale.

Following Institutional Animal Care and Use Committee approval under the protocol number of 10-2048A and informed consent from Weld County Humane Society, 28 clinically healthy female domestic shorthair cats scheduled for OHE were studied. Veterinary students under supervision of an experienced surgeon performed the OHE using a midline approach. Cats were random allocated in two groups: one group received hydromorphone^g^ (16 cats; 2.3 ± 0.9 kg; 8.5 ± 4.2 months) and the other fentanyl^h^ (12 cats; 2.6 ± 0.9 kg; 11.4 ± 5.5 months). The animals in the hydromorphone group were premedicated with hydromorphone (0.05 mg/kg) plus atropine^i^ (0.03 mg/kg) both SC, and at the end of the surgery received an additional dose of hydromorphone (0.025 mg/kg) as well as meloxicam^j^ (0.1 mg/kg), both SC. The animals in the fentanyl group were premedicated with atropine (0.03 mg/kg) SC, and received a dose of fentanyl (0.002 mg/kg) IV, just prior to surgery. In both groups anesthesia was induced with the combination of ketamine^k^ (5 mg/kg) and diazepam^l^ (0.25 mg/kg) IV and maintained with isoflurane^m^ in 100% of oxygen using a non-rebreathing system. If necessary to facilitate intubation a small dose of propofol^n^ (1 mg/kg) was administered. Three blinded observers recorded pain scores using the English version of the UNESP-Botucatu-MCPS and the interactive visual analogue scale (IVAS) in sequence. This was done 1 hour prior to surgery (before any medications, but after the cats had acclimatized to their environment for approximately 12 hours), and at 1, 2, 4, 6 and 24 hours after recovery from anesthesia. A single individual that was not involved in the pain assessment interacted with the cats (opened the cage, called by name, stroked the cat, played games with toys, offered food, and palpated the surgical area and abdomen). The three blinded evaluators observed behaviors at rest and during these interactions at the same time, but scored the cats independently and in the absence of any discussion.

Buprenorphine^o^ (0.02 mg/kg) IM and meloxicam (0.1 mg/kg) SC were administered for rescue analgesia, when two of the 3 evaluators agreed on the need for additional analgesic therapy based on their clinical experience. If subsequent additional rescue analgesia was deemed necessary, meloxicam was limited to a maximum dose of 0.2 mg/kg. Eight hours after the end of surgery, buprenorphine (0.02 mg/kg) oral transmucosally was administered to all cats, and meloxicam (0.1 mg/kg) SC administered to cats that had not previously received this drug.

### Construct validity by factor analysis

Principal components analysis with varimax rotation was performed to examine the underlying factor structure among items, and infer the dimensionality of the English version of the UNESP-Botucatu-MCPS. The identification of factors was based on the Kaiser criterion which suggests retaining all components with an eigenvalue >1 [49].

### Construct validity by known-group method

Known-group discrimination was used to assess if the total score and each subscale identified in the factor analysis were able to distinguish different severities of pain. The assessments of each observer were considered separately. Statistical differences were determined by Mann–Whitney test, with significance level of 5%.

### Internal consistency

Cronbach’s alpha coefficient [50] was used to assess the internal consistency of the English version of the UNESP-Botucatu-MCPS. The coefficient was calculated for both the overall scale and each subscale identified by factor analysis. Values for the Cronbach’s α coefficient > 0.7 were considered acceptable [27].

### Concurrent validity (criterion validation)

This was assessed by comparing the pain scores determined by the English version of the UNESP-Botucatu-MCPS with the pain scores registered by IVAS. A Spearman rank correlation coefficient was calculated for each observer separately.

### Inter-rater reliability

It was evaluated by ICC two-way random model and absolute agreement. The coefficient was calculated for each item of the scale at all time points. The results were interpreted by Altman’s classification [46].

## Endnotes

^a^ Propovan®

^b^ Fentanest®

^c^ Isoforine®

^d^ Dimorf® (Cristália Produtos Químicos Farmacêuticos Ltda.; Itapira, SP, Brazil)

^e^ Ketofen® (Merial Saúde Animal Ltda.; Paulínia, SP, Brazil)

^f^ Novalgina® (Sanofi-Aventis Farmacêutica Ltda.; Suzano, SP, Brazil)

^g^ Hydromorphone (Baxter Healthcare Corporation; Deerfield, IL, USA)

^h^ Fentanyl (Hospira; Lake Forest, IL, USA)

^i^ Atropine sulfate (Vedco Inc.; St. Joseph, MO, USA)

^j^ Metacam® (Boehringer Ingelheim Vetmedica Inc.; St. Joseph, MO, USA)

^k^ Ketaset® (Fort Dodge; Fort Dodge, IA, USA)

^l^ Diazepam (Hospira; Lake Forest, IL, USA)

^m^ Isoflurane (USP - Piramal Healthcare Ltd.; Andhra Pradesh, India)

^n^ Propoflo® (Abbott Laboratories; Chicago, IL, USA)

^o^ Buprenex® (Reckitt Benckiser Healthcare Ltd.; Hull, England, UK)

## Abbreviations

ACVA: American college of veterinary anesthesiology; AUC: Area under the curve; CI: Confidence interval; ICC: Intra-class correlation coefficient; NRS: Numerical rating scale; OHE: Ovariohysterectomy; ROC: Receiver operating characteristic; SDS: Simple descriptive scale; IVAS: Interactive visual analogue scale; MCPS: Multidimensional composite pain scale.

## Competing interests

The authors declare that they have no competing interests.

## Authors’ contributions

JTB conceived the study, carried out the animal experiment for video recording, prepared the DVDs, coordinated the clinical phase of the study (animal live) in the target culture, performed the statistical analysis and drafted the manuscript. KRM participated in the design of the study, performed the video analysis, supervised the clinical phase of the study (animal live) in an English-speaking country, and revised the final manuscript. SPLL participated in the design of the study, supervised the animal experiment for video recording, and assisted in revising the final manuscript. BDW performed the video analysis. SN, JA and PRV performed the video analysis and carried out the clinical phase (animal live) of the study in the target culture. CRP participated in the design of the study and supervised the statistical analysis. All authors read and approved the final manuscript.

## Authors’ information

JTB (DVM, PhD); KRM (DVM, Diplomate ACVA); SPLL (DVM, PhD, Diplomate ECVA); BDW (DVM, Diplomate ACVA); SN (DVM); JA (AAS, BS); PRV (AAS, CVT); CRP (BMath, PhD).
